# What to watch: Practical considerations and strategies for using YouTube for research

**DOI:** 10.1177/20552076221123707

**Published:** 2022-09-09

**Authors:** Wuyou Sui, Anna Sui, Ryan E Rhodes

**Affiliations:** 1 Department of Exercise Science, Physical and Health Education, 8205 University of Victoria, Victoria, Canada; 2 Department of Health and Rehabilitation Sciences, 6221 Western University, London, Canada

**Keywords:** YouTube, social media, methodology, big data, tutorial

## Abstract

YouTube is the second-most visited webpage in the world and boasts over 2 billion users and 500 h of videos uploaded every hour. Despite this popularity, relatively few articles have discussed the practical use of searching and YouTube as a research tool and source of data. The purpose of our paper is to propose a step-by-step schematic for utilizing the YouTube platform. Our discussions include (a) when/whether to use YouTube for research; (b) selecting an appropriate research design; (c) how to search for YouTube data; (d) what data can be pulled from YouTube; and (e) the contextual limitations for interpreting YouTube data. Further, we provide practical strategies and considerations when searching, collecting, or interpreting YouTube data. These discussions are informed by our own work using the YouTube platform. Effective methods used to search for YouTube data are likely to extend beyond simply searching the platform itself; the search strategy and search results themselves should also be documented. While not exhaustive, we feel these considerations and strategies present themselves as a conceptual foothold for future research using the YouTube platform.

## Background

Since their introduction, social media platforms, such as Twitter, Instagram, and Facebook, have been topics of interest to researchers as both research tools^1,3^ and as sources of data.^4,6^ As research tools, these platforms can be leveraged to advertise studies to targeted or diverse populations,^7,9^ procure representative responses and feedback from said populations,^10^ and be used as a means of intervention delivery.^11,13^ As sources of data, these platforms provide a wealth of freely accessible visual, textual, and metrics data by which to examine and visualize trends,^14^ analyse content and communities,^15^ and observe culture.^16^ Finally, the entirely participatory and user-generated nature of the content of social media provides a near-constant influx of new data from billions of users. For example, YouTube has seen a surge in recent usage, in part due to the coronavirus disease 2019 (COVID-19) pandemic,^12, 17, 18^ as well as through use as a resource for COVID-19 health information.^12, 19, 20^

YouTube's advantage is the global reach and popularity it has achieved. YouTube is owned by Google and is the most popular video-hosting site in the world, boasting 2 billion users (i.e., nearly 1/3 of all internet users;^21^ and over 500 h of content uploaded every minute,^22^ and over 1 billion hours of videos watched per day.^23^ Previous research has leveraged this data for content and thematic analyses,^24, 25^ development of instruments,^26^ large-scale interventions,^27^ and commentary on YouTube itself as a phenomenon.^28^

YouTube's popularity as a platform has resulted in handbooks and book chapters detailing how the platform may be leveraged for research.^29, 30^ However, the information provided within some of these resources is general and conceptual, rather than specific and actionable (e.g., where/how to search for videos/channels). The Second International Handbook for Internet Research^30^ reviews the ways that YouTube has been used for science and medicine but does not outline any specific strategies for practically using the platform for research. On the other hand, the SAGE Handbook of Social Media Research Methods^29^ does provide a detailed, general guide for conducting social media research and data collection/analysis. However, some nuances and specifics for conducting this research with YouTube are absent. Among a number of recently published papers utilizing YouTube for health research,^20,31,34^ no two studies utilized the same combination of methods for (a) searching for videos or creators, (b) selecting which videos or creators (and subsequent videos) were relevant to their research question, or (c) determining the data or metrics to be taken from the selected videos. Even a recently published research methods case report on vaccine safety and YouTube^35^ denotes a wide range of search methods for YouTube but does not offer any practical implication for how to address this. These inconsistencies highlight the potential issues for replication of these works; common approaches for searching and collating YouTube data are also key for the synthesis and reporting of results.

**Figure 1.**
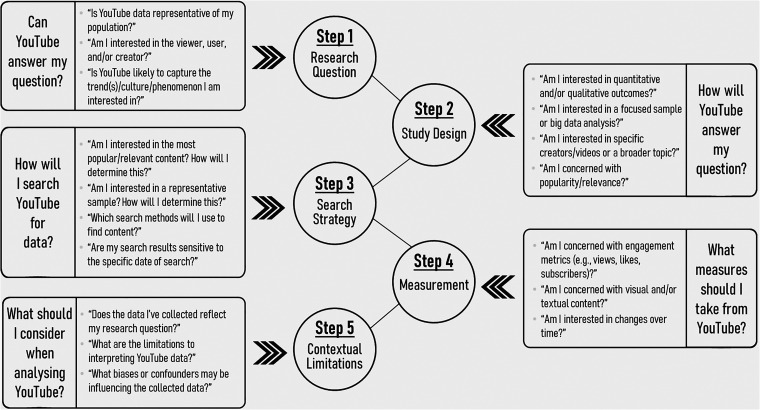
Step-by-step schematic for conducting research with YouTube.

In this paper, we provide a conceptual schematic by which future research utilizing YouTube data can build from. We also discuss challenges, considerations and recommendations for both quantitative and qualitative researchers seeking to leverage the YouTube platform as both a data collection tool and an open source of data; these discussions are conjointly mapped onto the step-by-step table and schematic (see Table 1 and Figure 1, respectively) that researchers can use to conceptualize, design, and conduct their own research using the YouTube platform. We begin our discussion with a brief overview of who uses YouTube; how YouTube data has been and can be used for research; followed by a summary of the forms of data and metrics that can be acquired from YouTube; suggestions for how to conduct searches for YouTube videos and creators, and lastly, contextual considerations when using the platform.

**Table 1.** Step-by-step table for conducting research with YouTube.

	Step 1: Research question ‘Is the YouTube platform appropriate to answer the research question?’	Step 2: Study design ‘Is the study design congruent with the research question?’	Step 3: Search strategy ‘Is the search strategy robust enough to find all relevant data?’	Step 4: Measurement ‘Do the outcomes align with the research question?’	Step 5: Contextual limitations ‘What should be considered when interpreting the data?’
Considerations	❏ Data needed to answer the research question can be found on the YouTube platform ❏ YouTube data is likely to be representative/valid for the research question	❏ Study design reflects the type of data to be extracted (i.e., qualitative, quantitative) ❏ Parameters of the search (e.g., channel/video selection criteria) reflect the research question ❏ Planned analyses align with the research question	❏ Method(s) of searching (e.g., YouTube, external websites, YouTube API) are likely to capture desired content ❏ Data selection criteria reflect the research question (e.g., channel popularity = composite of views and likes and subscribers) ❏ Search date, terms, results, and data extracted are recorded ❏ Browser cookies are cleared and/or private browsing/incognito account is being used	❏ Data collected is congruent with research question (e.g., visual and textual data for content analyses) ❏ Extraction date for engagement metrics is recorded (if taken) ❏ Methods for extracting/coding visual and textual data are explicit	❏ Sources of bias and/or confounders are minimized or accounted for ❏ Data extracted are interpreted within the context of how individuals use YouTube (i.e., viewers/users/creators) ❏ Interpretation(s) of results are situated within the appropriate temporal period

These discussions are informed, in part, by our own work utilizing YouTube data. Briefly, our own work explored longitudinal trends in home workout videos on YouTube during the COVID-19 pandemic, as well as visual and textual analyses of the most popular home workout and fitness creators’ videos. YouTube reported global average daily views of videos with ‘workout at home’ in the title increased by over 200% since March 15, 2020 (compared with the rest of the year prior^36, 37^). As such, we were interested in whether this surge of engagement with home workout videos would persist, given that engagement with traditional informal exercise programmes (e.g., gym membership) demonstrates a consistent sharp drop-off.^38^ Additionally, we conducted visual/textual analyses for the use of behaviour change techniques^39^ and other elements among the 15 most popular fitness channels on YouTube.^40^ Juxtaposition of our research onto the proposed schematic is detailed in Table 2.

**Table 2.** Qualitative and quantitative examples for conducting research with YouTube.

	Step 1: Research question ‘Is the YouTube platform appropriate to answer the research question?’	Step 2: Study design ‘Is the study design congruent with the research question?’	Step 3: Search strategy ‘Is the search strategy robust enough to find all relevant data?’	Step 4: Measurement ‘Do the outcomes align with the research question?’	Step 5: Contextual limitations ‘What should be considered when interpreting the data?’
Qualitative example: Content analysis	The purpose of this study was to analyse the ways in which visual and verbal content is used to shape ideas around fitness, fitness goals, and ‘health’.	A visual and textual analysis was used to explore content of the most popular fitness videos of the most popular fitness creators on YouTube Visual and textual content, including screenshots of the workout videos were extracted into NVivo 12 Pro where they were coded	Searches through Google including ‘Top’ Lists and through YouTube for popular fitness channels Channels with ≥1 million subscribers were screened with socialblade.com for popularity rankings and rankings of individual videos within each channel The top 15 channels were used, with the top 5 ‘most relevant’ videos (according to socialblade.com) extracted	Visual and textual data were extracted including: video screenshots, video descriptions, and YouTube generated video transcripts	Findings may be only representative of the YouTube platform and not of other sites of the fitness community The culture of other workout channels and/or videos may not be represented by our sample
Quantitative example: Longitudinal trends	The purpose of this study was to explore the pattern of engagement of YouTube fitness channels that posted either daily or programme-based fitness videos since the beginning of the coronavirus disease 2019 (COVID-19) pandemic.	Longitudinal study design was employed to describe temporal trends through changes in engagement metrics (quantitative) Multi-level models were planned for each engagement metric (except subscribers)	Searches on YouTube and through Google for combinations of search terms (e.g., ‘Daily’, ‘Workout’, ‘Exercise’, ‘Program’, ‘Fitness’, ‘At-Home’, ‘quarantine’, ‘lockdown’, and ‘COVID-19’ (and ‘YouTube’ for Google)) Channels were individually screened for posting daily or programme-based videos	Engagement metrics (i.e., views, likes, comments, subscribers) were extracted from videos on June 26, 2020 and July 8, 2020	Accounted for a channel's number of subscribers as a moderator Unclear how engagement with videos translates to actual exercise behaviour Limited sample of YouTube fitness videos

## Who uses YouTube?

**Figure 2.**
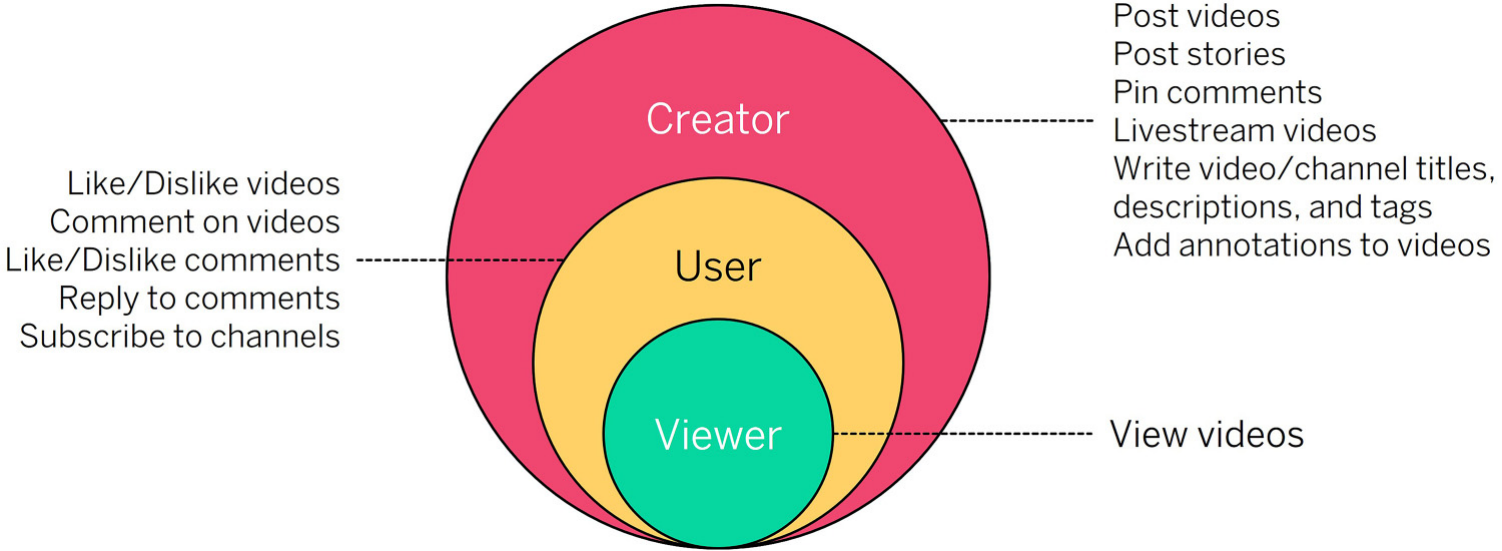
The different categories of engagement with YouTube and how they can interact with the YouTube platform.

In this section, we overview the differing types or levels of engagement that individuals can have with the YouTube platform. These data and metrics can be categorized conceptually into one of the three types of individuals engaging with YouTube: the viewer, the user, and the creator (see Figure 2).

### The viewer

The viewer describes an individual who engages with YouTube solely through watching videos, The smallest/lowest form of engagement that an individual can perform is the view. This category of ‘user’ also represents the broadest and least specific/descriptive form of engagement with YouTube.

### The user

The user describes an individual whose potential for engagement with the YouTube platform extends beyond simply watching videos and includes the numerous actions they can take via their Google account, which include (but are not limited to): leaving likes/dislikes on videos, posting/liking/disliking/replying to comments, and subscribing to channels.^41^

### The creator or YouTuber

The creator has the highest degree of platform engagement. Creators typically post videos, write descriptions, and gather a following of viewers and subscribers that help them reach micro-celebrity status.^42^

## Steps 1 and 2: The research question and study design

The unique nature of the YouTube platform poses a variety of research opportunities for both quantitative and qualitative researchers. In its simplest form, a single YouTube video or creator can provide a wealth of research content. Previous work explored content from one sole creator to examine the ‘brand’ or ‘ethos’ of that specific channel and the appeal of this content to its viewer base.^43^ YouTube creators – like PewDiePie (38.03 million subscribers), who post videogame content and streams – have appeared in the Business Insider, New York Times, and Forbes magazine,^44^ can arguably shape public opinions, and entice social action. The rise in children aspiring to be a YouTuber or vlogger as a future career reflects the influence of creators.^45, 46^ The predominantly video-focussed nature of YouTube has also engendered an entirely novel phenomenon. ‘Viral videos’ or videos that generate the majority of their social views within a few days of posting ^47^ inherently rely on video-hosting platforms for their existence, which can be explored through statistical methods.^48, 49^ Modern trends like ‘What I do/eat in a day’, ‘A day in the life of…’, and ‘[Verb] with me’ are ripe for ethnographic and critical discourse analyses. Categories of videos, like vlogging, are filmed as a one-sided conversation with the viewer and have been the topic of ethnographic research ^50^ and commentary.^28^ These messages and content, which directly appeal to a creator's viewers, encourage the development of parasocial relationships between the creator and their viewers, which has been the interest of previous quantitative and qualitative studies.^34, 51^ For researchers, a useful question may be ‘Is YouTube likely to capture the trend(s), culture, and/or phenomenon I am interested in?’. For instance, researchers interested in the formation of parasocial relationships between viewers and creators may ask ‘How are the creators building and maintaining relationships with their viewers?’.

The perpetual existence of videos after they are posted allows for temporal analysis of YouTube data. Videos posted on YouTube remain on the platform indefinitely, unless otherwise flagged for violating community guidelines ^52^ or removed by the creator. Hence, they can be utilized as a ‘window to the past’ or as representatives of trends across social and cultural movements. These data lend themselves well to longitudinal analysis, like modelling trends or variation in engagement in response to an event; cohort studies with specific creators or videos; or simply descriptive studies examining changes in popularity or engagement over time. Qualitative temporal analyses of video content (e.g., content analysis, discourse analysis, thematic analysis), as well as broader community and/or culture of a video/creator (e.g., via comments) are possible, and can provide insight into phenomena such as ‘cancel culture’ ^53^ and ‘viral trends’. Previous work has examined trends over periods of a few months,^54^ to 2 years,^34^ and 10 years.^55^ Related questions to guide the research process may include: ‘Am I interested in changes in engagement of a single video/channel over time?’, ‘Am I interested in how a topic or content evolves across time or in response to an event?’, or ‘Am I interested in comparing some aspect of a topic (e.g., community/discourse/culture) between timepoints?’.

Studies that are less concerned with longitudinal changes or temporal trends should still be mindful of when videos of interest were posted. Since engagement with a video is not limited to the date a video was posted, users can continually engage with content, long after a video has been released. As social and political climates shift, older videos that contain a – now, unfavourable – message, may garner new negative comments and dislikes, and by proxy receive more views. If an older video acquires a windfall of a new engagement, or ‘goes viral’, new comments may reflect new and different perspectives or cultures. Considerations for the temporal flux of engagement with a video or channel of interest should be noted. Questions may include: ‘Is my topic/channel of interest controversial, likely to be or go “viral”, or experience volatile levels of engagement?’ or ‘Is the relevance/importance/content of my topic sensitive to a specific period in time?’.

Another important point to consider is the general demographic of YouTube users. Recent estimates suggest that 81% of U.S. adults used YouTube, reaching 95% of U.S. internet users between the ages of 18–29, 91% of users aged 30–49, 83% of users aged 50–64, and 49% of users aged 65 years or older.^17^ In terms of gender, this reach is fairly even, with an estimated 82% of U.S. men and 80% of U.S. women using the platform.^17^ Consistent with other social media platforms, younger age groups tend to skew YouTube's user base. While this broader user demographic is likely not representative of every video/channel/topic, actual viewer demographics are privy only to the channel creator. Preliminary examination into the type of audience a video/channel/topic is likely to garner is suggested to ensure alignment between the research question and the data to be collected. Questions such as: ‘Is my population of interest likely to use YouTube to watch/search/engage with my topic?’ or ‘Is data collected from these videos or creators likely to be representative of the population I am interested in?’.

Certain populations may be underrepresented by the YouTube platform, in part due to the nature of the YouTube algorithm. Almost one third of queer and non-binary content on YouTube can be demonetized (i.e., cut off from advertising revenue) simply for using LGBTQ2 + associated words such as ‘gay’ and ‘lesbian’,^56^ which may discourage this content from being uploaded. An ongoing lawsuit against the platform alleges that BIPOC content was repeatedly removed from YouTube without an explanation.^57^ The ongoing complaints and lawsuits illustrate that the YouTube algorithm is not an apolitical tool and can come laden with prejudice. Researchers should be cognisant of the social and political dynamics that play out across the YouTube platform and formulate their research questions accordingly.

## Step 3: Search strategy

Searching for content on YouTube presents as its own unique challenge, especially for research purposes, where the replicability and notation of the research process is paramount. Over 500 h of content are uploaded to YouTube every minute.^22^ This sheer amount of new content can make it difficult to establish a clear picture of the scope of searched/collected data, making it difficult to determine what might constitute a representative sample. Previous studies have attempted to remedy this issue by selecting every *x* video to illustrate representativeness ^25^ or sorting by the most viewed videos.^58^ However, methods like these must be approached with caution, as the YouTube algorithm and how ‘popularity’ or ‘relevance’ of a video is determined can bias or confound searches.

The YouTube algorithm, among other things, determines which videos appear when a search is conducted on the platform. The algorithm ‘curates’ a series of videos to display based on numerous factors, including the ‘popularity’ of a video, a user's previously watched videos, the location of the user, and the specific day/time the search was performed. The algorithm is a ‘black box’, in that the specifics for which videos are displayed – and the order in which they are displayed in – are unknown to the searcher, rendering replications of searches near impossible.

Searching for videos/channels on YouTube may require a more complex approach than simply using the search bar. Below, we highlight the benefits and considerations to three distinct approaches to searching for YouTube data. Whether these approaches are used individually or in concert are up to the discretion of the researcher and/or the nature of the research question.

### The YouTube platform

Integrated into the YouTube webpage and app is the YouTube search bar into which keywords or phrases are typically entered. By default, search results are sorted by ‘relevance’, and include both videos and channels. Videos can be further sorted by ‘Upload date’, ‘View count’, and ‘Rating’, and further filtered by ‘Upload Date’ (i.e., Last hour, Today, This week, This month, This year), ‘Type’ (i.e., Video, Channel, Playlist, Movie), ‘Duration’ (i.e., Under 4 min, 4–20 min, Over 20 min), and ‘Features’ which includes options like location, HD, and subtitles/CC. The use of some operators can also help narrow searches ^59^: quotations will search for the exact search string; the plus operator (i.e., + [word]) forces results to include the specific word; similarly, the minus operator (i.e., − [word]) excludes results with the specific word; the pipe operator (i.e., [word1]|[word2]) returns results with either word; and the wildcard operator (i.e., *) will replace at least one word in a query. Hashtags (e.g., #fitness) can also be searched. Notably, while the number of results of a search used to appear with the search results, they no longer do as of the writing of this manuscript.

Beyond the search bar, YouTube also ranks and recommends videos based on browser history, cookies, previous videos watched by the viewer, user location, and new content, among others. However, how these videos are selected is difficult to track and the appropriateness of these videos is debated.^60^ More consistent across users are YouTube's trending lists, where the top 50 videos for categories of ‘Now’, ‘Music’, ‘Games’, and ‘Movies’ across the site are listed; however, these lists are subject to change daily, if not hourly.

Researchers should take pre-emptive steps to promote consistency and replicability of their searches. Searches using YouTube should be done with browser history and cookies cleared and on an incognito account or no account, so that these factors minimally influence search results. Location of the searcher (i.e., country of IP address) may also be relevant to report. When searches concern the most popular or relevant videos, sorting by ‘relevance’ appears to be the best choice, however these results should be interpreted cautiously and ideally in tandem with other search methods. Record of (a) what search terms and operators were used, (b) when the search was conducted, and (c) which videos/channels were extracted and from where (e.g., search query, recommended videos, etc.) specifically are crucial, as replication of the exact search results queried is very unlikely.

While this method of searching may give a general picture of the videos/channels that may show up in a given topic, how closely these results and recommendations represent what is actually seen by users ^60^ or the quality/quantity of the content on the platform is debatable and should be taken into account. Changes to the YouTube algorithm in 2019 have reduced viewership of what YouTube calls ‘borderline’ content (i.e., content that does not violate community guidelines, but is potentially harmful or misleading ^61^) by 70%. For research examining the presence or prevalence of these types of content (e.g., misinformation ^33^), relying on the YouTube algorithm may present an inaccurate picture of these contents. This method of searching may still impart a pseudo-representative sample of videos for a specific query (for the specific date of search); as such, studies seeking to use a ‘representative sample’ of videos should use the videos in the order they are presented, rather than taking every *n*th video, as the video order presented is more in line with what viewers are likely to see as well.

### External websites and search engines

The capability of external websites and search engines to search for YouTube data should be strongly considered by researchers looking to use YouTube data. These sources offer a more flexible and targeted way of searching and can often provide data-backed choices for channel or video selection. The Google search engine, for example, can be used as a direct alternative to the YouTube search engine, simply by adding ‘site:youtube.com’ into the search query. Moreover, searches into Google with the object ‘YouTube’ will often return curated or compiled lists of popular channels or videos for a particular topic. Similar to the YouTube search engine, these searches should ideally be completed with cookies cleared, browser history erased, and from an incognito account.

Aside from Google and other search engines, there are also webpages dedicated to tracking and ranking the metrics of social media platforms, like YouTube. One webpage we have used and will recommend is ‘Socialblade.com’, a social media analytics website. On Social Blade, every YouTube channel is assigned a rank and a grade based on a variety of metrics – including video views, subscribers – that signal the current popularity of a channel.^62^ In this way, channels can be compared to each other. Further, each channel is associated with four top 50 video lists: latest, most viewed, highest rated, and most relevant. The ‘most relevant’ list specifically can help researchers to determine which of a creator's videos are most popular at that moment, instead of relying solely on views or likes, which may introduce bias. Other metrics like country and similar channels can help to localize, globalize, and/or compare channels for selection. More generally, Social Blade also curates Top lists for specific topics (e.g., Education, Music, News & Politics), YouTube channels, and specific countries (e.g., Canada, Germany). Ideally, these search engines and webpages are used in concert, along with the YouTube search engine.

### The YouTube data application processing interface

One shared characteristic among the previously described search methods is that the exact search results are likely irreplicable by other researchers. Owing to the ever shifting and growing landscape of YouTube data, queried results using the same search strategy are likely to look very different. Further, the limit to how much data can be extracted is limited by human searching. However, an alternative search method, the YouTube Data Application Processing Interface (API), can return hundreds of relatively consistent search results based on a search query. Briefly, the YouTube Data API allows for integration of YouTube functionality into external webpages. Of relevance is the search function of the YouTube data API,^63^ which can return YouTube data that fit numerous specified parameters (e.g., location, published before/after, video type/duration). These results are returned in tabular form, with searches returned as code that can be used by other researchers within the API, akin to a search strategy in an academic database. We note that the use of the API involves a learning curve for those uninitiated in computer programming, and that we do not claim to be experts in this specific methodology. Use of the API may also best suit ‘big data’ approaches to leveraging YouTube. Indeed, multiple methods of sampling from the YouTube API have been documented by previous research in this field.^5, 64, 65^ However, there are resources available for navigating the API online and on YouTube. A simplified tool for extracting data from the API developed by Prof. Rieder called the YouTube Data Tools ^66^ offers a more user-friendly alternative for calling the API. Another tool – the Mozdeh Big Data Text Analysis programme, developed by a group at the University of Wolverhampton ^67^ – leverages the YouTube API to gather video comments which can then be mined for word associations and sentiment and filtered by keywords/likes/gender/etc., among other functions.

## Step 4: Measurement

The YouTube platform is rich in the types of data that can be extracted, both quantitative and qualitative. Previous research by Giglietto et al, ^6^ has categorized data via interactions: audience interactions (i.e., views), social interactions (i.e., likes and comments), and platform interactions (i.e., meta-data). We build upon the work of Giglietto et al,^6^ proposing four broader types of data: engagement metrics, video/channel characteristics, textual data, and visual data. Below we describe these forms of data.

### Engagement metrics

We define engagement metrics as data that quantifiably represent the interactions that viewers and users have with creators and the YouTube platform. These typically include video views, video likes/dislikes, comments/replies to comments, comment likes/dislikes, and subscriber count.

#### Views

YouTube counts a ‘view’ when (a) an individual intentionally initiates watching a video (i.e., clicks the play button or the video thumbnail) and (b) watches at least 30 s of the video.^68^ This watch time threshold is considerably longer than other platforms (e.g., Facebook and Instagram = 3 s, Twitter = 2 s; ^68^ as such, views on YouTube are likely more robust measures of viewership, compared to other social media platforms. As a metric, views offer the largest estimate of engagement with a video. Moreover, since no account is required by the viewer for a video to register a view, this data also offers the broadest measure of engagement with a video. As such, views are typically an outcome of interest for studies utilizing YouTube data to examine popularity or engagement (e.g., Literary works^69,73^). Further, views may be counted from outside the YouTube webpage. For example, embedded videos on other webpages count as views. Hence, views may be more representative of engagement with the specific video, rather than the YouTube platform itself. Views can further be used as search criteria; whether to determine the most popular videos,^71^ to identify the most popular videos for a creator,^74^ or both.

#### Likes/dislikes

Whereas a view is passive in that no further action from the viewer is necessary beyond simply clicking on the video, likes represent the smallest form of active engagement with a video (or comment). Further, similar to the view, which is the smallest form of interaction with a video, likes/dislikes are the smallest forms of interaction with a video creator that a user can have. However, owing to the need for an account to leave a like, the number of likes a video has is typically far lower than the number of views a video has. Moreover, the number of dislikes is also typically much lower than the number of likes a video receives.

YouTube users can leave a like or dislike on a video which is also saved to a user's account under a ‘liked videos’ playlist YouTube depicts the ratio of likes to dislikes on a video through (a) a number next to a thumbs up and thumbs down icon, respectively; (b) a dark grey/light grey bar, whereby the dark grey bar represents the percentage of likes and the light grey represents the percentage of dislikes; and (c) the exact number of likes/dislikes if the mouse is hovered over the bar.

#### Comments

According to YouTube, comments allow creators to ‘get direct feedback from your viewers, answer their questions, and overall create a community and conversation around [their] videos’.^75^ Accordingly, some studies have solely examined comments as a source of data.^76,78^ Functionally, YouTube users can leave comments on a video, as well as reply to comments, and leave likes/dislikes on comments. The total number of comments is displayed underneath the video, as are the replies to a comment. Comments can be sorted by either ‘Top comments’ or ‘Newest first’. Creators of a video can also ‘pin’ either user comments or their own comments to show up first and can also ‘heart’ specific comments as a way of promotion, which are demarked from other comments with a small badge with a heart underneath the comment. Creators can further delete comments or hold comments with certain words for review or even turn off comments completely.^79^

### Video/channel characteristics

Separate from the interactions that viewers, users, and creators engage in with videos and each other, there is also data that can be drawn as a function of the video or channel itself. For creators/channels, the total number of video uploads, video length, channel start date, and video posting frequency can offer an estimate of a creator's activity on the platform. Similarly, the video post-date and length of video can be used as comparators between videos.

### Visual content

Within the YouTube platform, several elements of video content can be extracted for analysis. First, the videos themselves can be analysed. This can be done by analysing the video as a whole or by extracting screenshots. Notably, complete YouTube videos *can* be extracted with screen capture software or converted to other file formats for download. Within the video, additional visual elements such as banners (i.e., annotations, instructions, and links to other videos) can be extracted for analysis. Videos can contain several elements for analysis including human elements such as people who appear in the video, environmental elements such as the set-pieces of the video, and camera angles – the *way* a video is shot. In this way, the creator themselves can be the subject of the visual content or data (e.g., ethnicity, age, gender, etc.). Video thumbnails, when used in research, can also be of interest as they provide a snapshot into the content of the video at a glance. As well, a series of such snapshots can be seen in the form of ‘recommended videos’, which is a reel appearing to the right of the observed video as to suggest videos of a similar nature.

### Textual content

From the perspective of language and discourse, YouTube videos can be transcribed, coded, and analysed according to the research question. As of the writing of this paper, YouTube has an ‘open transcript’ function that displays a transcript that can be extracted, with or without timestamps, which greatly facilitates the process of extracting video textual data. In addition, the textual content can also be added to accompany videos. Creators can do this in several ways, including video descriptions, video titles, about pages of the channel, and video tags. Additional textual data links subscribers and viewers to the creators’ other platforms, such as Patreon and Instagram, and to promotes additional materials. These self-promotional messages encourage a creator's viewers and users to engage with the creator both inside and outside of the YouTube platform, which may manifest as features in future videos. YouTube comments are also a lucrative source of textual data as they reflect the opinions of the community within a channel. As such, comments can be used an indicator of the discourse around a topic, the culture of a community, or as textual reference for the likes/dislikes of a video. Alternatively, if the video and its content closer reflect the creator, then comments closer resemble the perspectives, opinions, and feelings of the community. Overall, much of this textual data provides an opportunity for YouTubers to engage further with their subscriber and viewer base outside of the video, and to garner more visibility through their other platforms.

## Step 5: Contextual limitations and considerations

Once appropriate means of searching and selecting YouTube data have been conducted, some considerations should be accounted for when extracting and interpreting data. As previously mentioned, views are typically an outcome of interest for studies examining popularity or engagement (e.g.,^69,71^ By the very nature of the YouTube platform, videos with an earlier post-date have more time, and thus more potential, to accrue views. Given that only current view counts are available for a video (unless you are the video owner), it can be difficult to track trends in viewership. Further, views on a video may not be representative of the popularity of a creator, as compared to the total views a creator has accrued. The phenomenon of a viral video exemplifies this well, whereby the popularity of a single video drives engagement with the creator, rather than the popularity of the creator driving consistent engagement with all their content.

Conversely, given that an action must be taken by the user to leave a like or dislike, these metrics reveal more of the affective leanings that a video garners. When considering a metric for popularity, likes/dislikes should be considered in tandem with views and upload date. The music video for Justin Beiber's song ‘Baby’; despite (or perhaps due to) being the second most disliked videos on YouTube with 12 million dislikes, the video has also garnered over 2.5 billion views.^80^ Notably, views (or other video metrics) may not be of interest at all. Ethnographic studies and content analyses of specific videos may not require views as either a search criterion or collect views as an outcome of interest (e.g., the work of McDaniel^81^).

Research involving comments should also be evaluated carefully as they may represent a ‘loud minority’ of the community and should be taken with a grain of salt, particularly for more polarizing or controversial topics (e.g., politics, religion;^82^ Further, the ‘top comments’ are not ranked by most likes, most replies, or date posted, but likely some combination therein. As such, the top comments may not reflect the most recent discourse or opinion surrounding a video, unlike sorting by ‘newest first’.

Researchers should also be wary of interpreting YouTube data. For example, in our study of online workout videos, we gauged the popularity of the videos based on the channel subscribers and the individual video views. We could also explore other ways in which viewers were engaging with the content such as using the comments section. However, we could not conclude that all the viewers were actually following the workouts at home. Hence, YouTube data informs us of a limited scope of engagement and researchers should be careful about the interpretation of this data as indicative of viewers behaviour and/or associated outcomes of interest

Finally, researchers need to consider the rapid changes that occur on the YouTube platform within mere days and sometimes hours. New additions to the platform can substantially change the ways in which people can interact with it and even those changes are often removed after short trial periods. Videos themselves can often be banned due to copyright infringement or for violating YouTube posting guidelines. Therefore, research practices with YouTube needs to be hands-on and require more active engagement by the researcher than other methods and modes of digital research.

## Conclusion

Our paper aimed to highlight some of the considerations and offer some practical strategies when using YouTube for research, as well as put forth a conceptual schematic for guiding research using YouTube. The potential for YouTube as a research tool and source of data is considerable; however, it is clear that several considerations must be taken into account when deciding on whether YouTube is appropriate for a research question; determining a study design; determining which/how much data to extract; creating a robust search strategy for YouTube data; and how to interpret/use this data. Alignment of research purpose, methodology (i.e., qualitative, quantitative), design, population, and outcome(s) or data of interest should be cogent. Further, methods used to search for YouTube data are likely to extend beyond simply searching the platform itself and should be documented along with search results. Finally, interpretation of these data should be done cautiously, as the representativeness of (1) views as a measure of popularity, (2) the engagement of users (i.e., likes, comments), and (3) the content of creators may not be reflective of trends or behaviour outside of the platform. While not exhaustive, we feel these considerations, strategies, and the proposed schematic present as a conceptual foothold for future research using the YouTube platform.

## References

[1] Konijn EA Veldhuis J Plaisier XS . YouTube As a research tool: three approaches. Cyberpsychol Behav Soc Netw 2013; 16: 695–701. 23659721 10.1089/cyber.2012.0357

[2] Vitak J . Facebook as a Research Tool in the Social and Computer Sciences. In: The SAGE Handbook of Social Media Research Methods. 1 Oliver’s Yard, 55 City Road London EC1Y 1SP: SAGE Publications Ltd; 2016. p. 627–42.

[3] Gelinas L Pierce R Winkler S , et al. Using Social Media as a research recruitment tool: ethical issues and recommendations. Am J Bioeth 2017; 17: 3–14. 10.1080/15265161.2016.1276644 PMC5324729 28207365

[4] Pace S . YouTube: an opportunity for consumer narrative analysis? Cova, editor. Qual Market Res Int J 2008; 11: 213–226.

[5] Karkulahti O Kangasharju J . Youtube Revisited: On the Importance of Correct Measurement Methodology. In 2015. p. 17–30.

[6] Giglietto F Rossi L Bennato D . The open laboratory: limits and possibilities of using Facebook, Twitter, and YouTube as a research data source. J Technol Hum Serv 2012; 30: 145–159.

[7] Kent M Ellis K . People with disability and new disaster communications: access and the social media mash-up. Disabil Soc 2015; 30: 419–431.

[8] Blackwell L Hardy J Ammari T , et al. LGBT Parents and social Media. In: Proceedings of the 2016 CHI conference on human factors in computing systems. New York, NY, USA: ACM, 2016, pp.610–622.

[9] McClanahan A . The Downfalls of Performative White Allyship on Social Media in the #BlackLivesMatter Movement. Morgantown, WV: West Virginia University, 2021.

[10] Green M Bobrowicz A Ang CS . The lesbian, gay, bisexual and transgender community online: discussions of bullying and self-disclosure in YouTube videos. Behav Inf Technol 2015; 34: 704–712.

[11] Westerman D Spence PR Van Der Heide B . Social Media as information source: recency of updates and credibility of information. J Comput Mediat Commun 2014; 19: 171–183.

[12] Choukou MA Sanchez-Ramirez DC Pol M , et al. COVID-19 infodemic and digital health literacy in vulnerable populations: a scoping review. Digit Health 2022; 8: 205520762210769. Available from: http://journals.sagepub.com/doi/10.1177/20552076221076927. 10.1177/20552076221076927 PMC8874333 35223076

[13] Hong YA Yee S Bagchi P , et al. Social media-based intervention to promote HBV screening and liver cancer prevention among Korean Americans: results of a pilot study. Digit Health 2022; 8: 205520762210762. Available from: http://journals.sagepub.com/doi/10.1177/20552076221076257. 10.1177/20552076221076257 PMC8819816 35140979

[14] Schreck T Keim D . Visual analysis of Social Media data. Computer (Long Beach Calif) 2013; 46: 68–75.

[15] Shrestha A Kaati L Cohen K . Extreme adopters in digital communities. J Threat Assess Manage 2020; 7: 72–84.

[16] van Dijck J . The Culture of Connectivity. The Culture of Connectivity. Oxford University Press, 2013.

[17] Auxier B Anderson M . Social Media Use in 2021 [Internet]. Pew Research Center. 2021 [cited 2021 Aug 18]. Available from: https://www.pewresearch.org/internet/2021/04/07/social-media-use-in-2021/.

[18] Stewart R Madonsela A Tshabalala N , et al. The importance of social media users’ responses in tackling digital COVID-19 misinformation in Africa. Digit Health 2022; 8: 205520762210850. Available from: http://journals.sagepub.com/doi/10.1177/20552076221085070. 10.1177/20552076221085070 PMC8935564 35321021

[19] Auxier B Anderson M . Social Media Use in 2021 [Internet]. Pew Research Center. 2021 [cited 2021 Aug 17]. Available from: https://www.pewresearch.org/internet/2021/04/07/social-media-use-in-2021/.

[20] Li HOY Bailey A Huynh D , et al. YouTube As a source of information on COVID-19: a pandemic of misinformation? BMJ Global Health 2020; 5: e002604. Available from: 10.1136/bmjgh-2020-002604 PMC7228483 32409327

[21] Cohen L . Why marketers should care about the music industry’s latest transformation [Internet]. Think with Google. 2020 [cited 2021 Aug 18]. Available from: https://www.thinkwithgoogle.com/marketing-strategies/video/music-industry-changes/.

[22] Wojcicki S . YouTube at 15: My personal journey and the road ahead [Internet]. YouTube Official Blog. 2020 [cited 2021 Aug 19]. Available from: https://blog.youtube/news-and-events/youtube-at-15-my-personal-journey/.

[23] Goodrow C . You know what’s cool? A billion hours [Internet]. YouTube Official Blog. 2017 [cited 2021 Aug 18]. Available from: https://blog.youtube/news-and-events/you-know-whats-cool-billion-hours.

[24] Ratwatte P Mattacola E . An exploration of ‘fitspiration’ content on YouTube and its impacts on consumers. J Health Psychol 2021; 26: 935–946. 31190554 10.1177/1359105319854168

[25] Yoo JH Kim J . Obesity in the new Media: a content analysis of obesity videos on YouTube. Health Commun 2012; 27: 86–97. 21809934 10.1080/10410236.2011.569003

[26] Ryoo Y Yu H Han E . Political YouTube Channel Reputation (PYCR): development and validation of a multidimensional scale. Telemat Inform 2021; 61: 101606.

[27] Lutkenhaus RO Wang H Singhal A , et al. Using markers for digital engagement and social change: tracking meaningful narrative exchange in transmedia edutainment with text analytics techniques. Digit Health 2022; 8: 205520762211078. Available from: http://journals.sagepub.com/doi/10.1177/20552076221107892.

[28] Arthurs J Drakopoulou S Gandini A . Researching YouTube. Convergence 2018; 24: 3–15.

[29] Sloan L Quan-Haase A . The SAGE Handbook of Social Media Research Methods. 1 Oliver’s Yard, 55 City Road London EC1Y 1SP: Sage Publications Ltd, 2016.

[30] Hunsinger J Allen MM Klastrup L (eds). Second International Handbook of Internet Research. Dordrecht: Springer Netherlands, 2020.

[31] Al Mahmud A Le A Mubin O . Use of YouTube as a Source of Information for Quitting or Cutting Down Alcohol. Front Public Health 2021; 9: 6. 10.3389/fpubh.2021.787994 PMC8718438 34976933

[32] Çapan BŞ . YouTube As a source of information on space maintainers for parents and patients. Lavorgna L, editor. PLOS ONE 2021; 16: e0246431. 33571208 10.1371/journal.pone.0246431 PMC7877623

[33] Hussein E Juneja P Mitra T . Measuring misinformation in video search platforms: an audit study on YouTube. Proc ACM Hum Comput Interact 2020; 4: 1–27.

[34] Ferchaud A Grzeslo J Orme S , et al. Parasocial attributes and YouTube personalities: exploring content trends across the most subscribed YouTube channels. Comput Human Behav 2018; 80: 88–96.

[35] Basch C Basch C . Emerging Methods in Health-Related Social Media Research: A Case Study of Vaccine Safety and YouTube. 1 Oliver’s Yard, 55 City Road, London EC1Y 1SP United Kingdom: SAGE Publications Ltd, 2020.

[36] The Explainer: Workout At Home [Internet]. YouTube Culture & Trends. 2020 [cited 2021 Aug 18]. Available from: https://www.youtube.com/trends/articles/stay-home-workout-at-home/.

[37] Sui W Rush J Rhodes RE . Engagement with web-based fitness videos on YouTube and Instagram during the COVID-19 pandemic: longitudinal study. JMIR Formative Res 2022; 6: e25055. 10.2196/25055 PMC8906834 35258459

[38] Dishman RK . Exercise adherence research: future directions. Am J Health Promot 1988; 3: 52–56. 22206240 10.4278/0890-1171-3.1.52

[39] Michie S Richardson M Johnston M , et al. The behavior change technique taxonomy (v1) of 93 hierarchically clustered techniques: building an international consensus for the reporting of behavior change interventions. Ann Behav Med 2013; 46: 81–95. 23512568 10.1007/s12160-013-9486-6

[40] Sui W Morava A Tsang J , et al. Describing the use of behavior change techniques among the most popular home workout channels on YouTube: a quantitative content analysis. J Health Psychol 2022. Available from: 10.1177/13591053221074584. 35114825

[41] YouTube Help. Comment, subscribe, & connect with creators [Internet]. Google. 2021 [cited 2021 Aug 18]. Available from: https://support.google.com/youtube/topic/9257418?hl=en&ref_topic=9257500.

[42] Khamis S Ang L Welling R . Self-branding, ‘micro-celebrity’ and the rise of social Media influencers. Celebr Stud 2017; 8: 191–208.

[43] Schneider CJ . “I wish I could grow a full beard”: the Amateur Pogonotropher on the beardbrand YouTube channel. Cult Stud Crit Methodol 2020; 20: 295–306.

[44] Tassi P . PewDiePie Hits 100 Million Subscribers, And Surprisingly, YouTube Pays Him Tribute [Internet]. Forbes. 2019 [cited 2021 Aug 18]. Available from: https://www.forbes.com/sites/paultassi/2019/08/25/pewdiepie-hits-100-million-subscribers-and-surprisingly-youtube-pays-him-tribute/?sh=2d7ca93237f5.

[45] Chambers N Kashefpakdel ET Rehill J , et al. Drawing the future. 2018.

[46] The LEGO Group. LEGO Group Kicks Off Global Program To Inspire The Next Generation Of Space Explorers As NASA Celebrates 50 Years Of Moon Landing [Internet]. Cision US. 2019 [cited 2021 Aug 18]. Available from: https://www.prnewswire.com/news-releases/lego-group-kicks-off-global-program-to-inspire-the-next-generation-of-space-explorers-as-nasa-celebrates-50-years-of-moon-landing-300885423.html.

[47] Broxton T Interian Y Vaver J , et al. Catching a viral video. J Intell Inf Syst 2013; 40: 241–259.

[48] Jiang L Miao Y Yang Y , et al. Viral video style. In: Proceedings of international conference on multimedia retrieval. New York, NY, USA: ACM, 2014, pp.193–200.

[49] Burgess J. ‘All your chocolate rain are belonging to us?’: viral video, YouTube and the dynamics of participatory culture. In: Art in the global present. Sydney: University of Technology, 2014, pp.86–96.

[50] Hou M . Social media celebrity and the institutionalization of YouTube. Convergence 2019; 25: 534–553.

[51] Tolbert AN Drogos KL . Tweens’ Wishful Identification and Parasocial Relationships With YouTubers. Front Psychol 2019; 10: 15. 10.3389/fpsyg.2019.02781 PMC6928007 31920829

[52] Community Guidelines [Internet]. YouTube. 2021 [cited 2021 Aug 19]. Available from: https://www.youtube.com/howyoutubeworks/policies/community-guidelines/.

[53] Dodgson L . How Shane Dawson went from “King of YouTube” to the biggest fall from grace the platform has ever seen [Internet]. Insider. 2020 [cited 2021 Aug 19]. Available from: https://www.insider.com/how-shane-dawson-went-from-king-of-youtube-to-canceled-2020-7.

[54] Weaver AJ Zelenkauskaite A Samson L . The (non)Violent world of YouTube: content trends in web video. J Commun 2012; 62: 1065–1083.

[55] Hynes SM Ghahari S Forwell SJ . “Waiting for science to catch up with practice”: an examination of 10-year YouTube trends in discussions of chronic cerebral spinal venous insufficiency treatment for multiple sclerosis. Inform Health Soc Care 2019; 44: 327–337. 30913949 10.1080/17538157.2019.1582052

[56] Romano A . A group of YouTubers is trying to prove the site systematically demonetizes queer content [Internet]. Vox. 2019 [cited 2021 Aug 19]. Available from: https://www.vox.com/culture/2019/10/10/20893258/youtube-lgbtq-censorship-demonetization-nerd-city-algorithm-report.

[57] Albergotti R . Black creators sue YouTube, alleging racial discrimination. The Washington Post 2020 Jun 18.

[58] D’Souza RS D’Souza S Strand N , et al. YouTube As a source of medical information on the novel coronavirus 2019 disease (COVID-19) pandemic. Glob Public Health 2020; 15: 935–942. 32397870 10.1080/17441692.2020.1761426

[59] Stegner B . How to Search YouTube Like a Pro Using Advanced Search Operators [Internet]. MakeUseOf. 2020 [cited 2021 Aug 19]. Available from: https://www.makeuseof.com/tag/search-youtube-pro-google-advanced-operators/.

[60] Madrigal AC . How YouTube’s algorithm really works. The Atlantic. 2018 Nov.

[61] The YouTube Team. The Four Rs of Responsibility, Part 2: Raising authoritative content and reducing borderline content and harmful misinformation [Internet]. YouTube Official Blog. 2019 [cited 2021 Aug 19]. Available from: https://blog.youtube/inside-youtube/the-four-rs-of-responsibility-raise-and-reduce/.

[62] FREQUENTLY ASKED QUESTIONS (FAQ) [Internet]. Social Blade. 2021 [cited 2021 Aug 19]. Available from: https://socialblade.com/youtube/help.

[63] Search: list [Internet]. Google Developers. 2021 [cited 2021 Aug 19]. Available from: https://developers.google.com/youtube/v3/docs/search/list

[64] Malik H Tian Z . A framework for collecting YouTube meta-data. Procedia Comput Sci 2017; 113: 194–201.

[65] Bärtl M . YouTube Channels, uploads and views. Convergence 2018; 24: 16–32. Available from: http://journals.sagepub.com/doi/10.1177/1354856517736979.

[66] Rieder B . YouTube Data Tools (Version 1.22) [Internet]. 2015 [cited 2021 Aug 19]. Available from: https://tools.digitalmethods.net/netvizz/youtube/index.php.

[67] Statistical Cybermetrics Research Group. Mozdeh Big Data Text Analysis [Internet]. 2021 [cited 2021 Aug 19]. Available from: http://mozdeh.wlv.ac.uk/.

[68] Funk M . How Does YouTube Count Views? [Internet]. Tubics. 2020 [cited 2021 Aug 19]. Available from: https://www.tubics.com/blog/what-counts-as-a-view-on-youtube/.

[69] Figueiredo F Almeida JM Benevenuto F , et al. Does content determine information popularity in social media? In: Proceedings of the SIGCHI conference on human factors in computing systems. New York, NY, USA: ACM, 2014, pp.979–982.

[70] Schwemmer C Ziewiecki S . Social Media sellout: the increasing role of product promotion on YouTube. Social Media + Society 2018; 4: 205630511878672.

[71] Liikkanen LA Salovaara A . Music on YouTube: user engagement with traditional, user-appropriated and derivative videos. Comput Human Behav 2015; 50: 108–124.

[72] Yang S Brossard D Scheufele DA , et al. The science of YouTube: what factors influence user engagement with online science videos? PLOS ONE 2022; 17: e0267697. Available from: 35613095 10.1371/journal.pone.0267697 PMC9132274

[73] Zaila KE Osadchiy V Anderson AS , et al. Popularity and worldwide reach of targeted, evidence-based internet streaming video interventions focused on men’s health topics. Transl Androl Urol 2020; 9: 1374–1381. Available from: http://tau.amegroups.com/article/view/43369/html. 32676422 10.21037/tau-20-580 PMC7354348

[74] Maloney M Roberts S Caruso A . ‘Mmm … I love it, bro!’: performances of masculinity in YouTube gaming. New Media Society 2018; 20: 1697–1714.

[75] Comments overview [Internet]. YouTube Creator Academy. 2021 [cited 2021 Aug 19]. Available from: https://creatoracademy.youtube.com/page/lesson/connect-with-comments_overview_video#strategies-zippy-link-1.

[76] Teng S Khong KW Pahlevan Sharif S , et al. YouTube Video comments on healthy eating: descriptive and predictive analysis. JMIR Public Health Surveill 2020; 6: e19618. 33001036 10.2196/19618 PMC7563625

[77] Fernandez-Luque L Elahi N Grajales FJ 3^rd^. An analysis of personal medical information disclosed in YouTube videos created by patients with multiple sclerosis. Stud Health Technol Inform 2009; 150: 292–296. 19745316

[78] Kavitha KM Shetty A Abreo B , et al. Analysis and classification of user comments on YouTube videos. Procedia Comput Sci 2020; 177: 593–598. Available from: https://linkinghub.elsevier.com/retrieve/pii/S1877050920323553.

[79] Handle hurtful or inappropriate comments [Internet]. YouTube Creator Academy. 2021 [cited 2021 Aug 19]. Available from: https://creatoracademy.youtube.com/page/lesson/connect-with-comments_handle-inappropriate-comments_list?cid=connect-with-comments&hl=en.

[80] Bieber J . Justin Bieber - Baby (Official Music Video) ft. Ludacris [Internet]. YouTube. 2010 [cited 2021 Aug 19]. Available from: https://www.youtube.com/watch?v=kffacxfA7G4.

[81] McDaniel B . Popular music reaction videos: reactivity, creator labor, and the performance of listening online. New Media Society 2021; 23: 1624–1641.

[82] Siersdorfer S Chelaru S Nejdl W , et al. How useful are your comments? In: Proceedings of the 19th international conference on World wide web - WWW ’10. New York. New York, USA: ACM Press, 2010, p. 891.

